# Akshar Mitra: a multimodal integrated framework for early dyslexia detection

**DOI:** 10.3389/fdgth.2025.1726307

**Published:** 2025-11-28

**Authors:** Vibha Tiwari, Ocean Agarwal, Manya Sharma, Rashi Sahu, Radhika Babar, Rebakah Geddam, Muhammad Awais, Hemant Ghayvat

**Affiliations:** 1 Center for Artificial Intelligence, Madhav Institute of Technology and Science (Deemed University), Gwalior, India; 2 Department of Computer Science and Media Technology, Faculty of Technology, Linnaeus University, Växjö, Sweden; 3 Department of Computer Science, Madhav Institute of Technology and Science (Deemed University), Gwalior, India; 4 Center of Internet of Things, Madhav Institute of Technology and Science (Deemed University), Gwalior, India; 5 Unitedworld Institute of Technology, Karnavati University, Gujarat, India; 6 Department of Medical Physics, Memorial Sloan Kettering Cancer Center, New York, NY, United States

**Keywords:** dyslexia detection, multimodal framework, early screening, health technology, neurodevelopmental disorders, digital health, cognitive assessment

## Abstract

Developmental dyslexia is a prevalent neurobiological disorder affecting 10%–15% of children globally, yet it remains largely undiagnosed due to the inaccessibility of conventional assessments in resource-limited settings. Existing screening methods are further constrained by their reliance on unimodal data streams and the need for large, clinically-labeled datasets. This paper presents Akshar Mitra, a Multimodal Integrated Framework (MMF), a novel computational methodology designed for accessible and early dyslexia screening. The framework pioneers the integration of three low-cost, high-yield digital biomarkers derived from eye-tracking, speech, and handwriting analysis.The MMF is implemented through three modules: webcam-based eye-tracking for fixation and saccadic analysis, automated speech assessment for fluency metrics, and optical character recognition for handwriting error detection. Each module extracts 4–6 interpretable features (e.g., fixation regressions, word-error rate, character reversals) that are standardized via a shared data schema. These objective measures are augmented by a concise behavioral questionnaire to generate a holistic risk profile. Beyond screening, the system incorporates support tools, including a dyslexia-friendly reading interface with syllable-level highlighting, to foster user engagement and confidence.By creating a scalable, language-agnostic, and explainable system, this work offers a viable pathway to bridge the global dyslexia diagnostic gap. The MMF provides a transformative tool for proactive screening, facilitating early intervention and improving educational outcomes.

## 1 Introduction

Dyslexia (1, 2) is one of the most common learning disorders in children, yet also one of the most misunderstood, particularly in countries such as India. It is characterized by persistent difficulties in reading, spelling, and decoding words, despite adequate intelligence and educational opportunity (3). Globally, dyslexia affects an estimated 5%–15% of the population; in India, recent studies suggest that 10%–15% of school-aged children i.e., approximately 35–40 million individuals may exhibit dyslexic traits. Unfortunately, more than 80% of dyslexic children in India go undiagnosed and frequently being mislabelled as reckless or underachieving. Such delays in identification can have profound consequences, negatively impacting academic performance, self-esteem, and long-term emotional well-being (4, 25).

The core challenges in early identification stem from limited public awareness, pervasive social stigma, and the high cost of professional assessments (ranging from Rs.5,000 to Rs.30,000), which are primarily offered in urban centers. Most schools, specially in rural and semi-urban regions lack trained personnel and resources to detect learning disabilities at an early stage. Consequently, remedial interventions often occur only after years of struggle, when their efficacy is substantially diminished.

A firm consensus in neuroscience establishes dyslexia as a brain-based learning disorder with a neurobiological origin, completely separate from an individual’s general intelligence (5, 6). Neuroimaging research has supplied compelling evidence for structural and functional differences in the brains of individuals with dyslexia compared to typical readers (3). These observable phenomena provide the foundation for the digital biomarkers.

Various technological approaches have also been explored to facilitate the detection and intervention of dyslexia (7). Eye-tracking systems (8), for example, have been used to quantify reading behaviors such as fixation durations and regressions, which differ significantly in dyslexic readers (9). Deep learning–based handwriting analysis has demonstrated promise in detecting dysgraphia and related motor-writing symptoms (10). Similarly, speech-based methods have been employed to assess reading fluency and pronunciation patterns indicative of dyslexia.However, existing solutions are often hardware-dependent, costly, or limited to a single modality, and they seldom account for the socioeconomic diversity encountered in regions like India.

### 1.1 Contribution and novelty

To address these limitations, **Akshar Mitra**-a unified, AI-powered platform for early dyslexia detection and support-is proposed in this work. Akshar Mitra integrates multiple modalities into a single, accessible system, including:

1. Eye-Tracking Analysis, which captures real-time fixation and saccade metrics via webcam while simultaneously speaking the text through Speech-to-Text Processing, which computes prosodic and fluency features from transcribed reading passages.
2. Handwriting Recognition, using OCR-based extraction of written text followed by symbol-level error analysis
3. Behavioral Quiz, a parental or self-report questionnaire that captures observable dyslexia-related behaviors at home.

**Figure 1:**
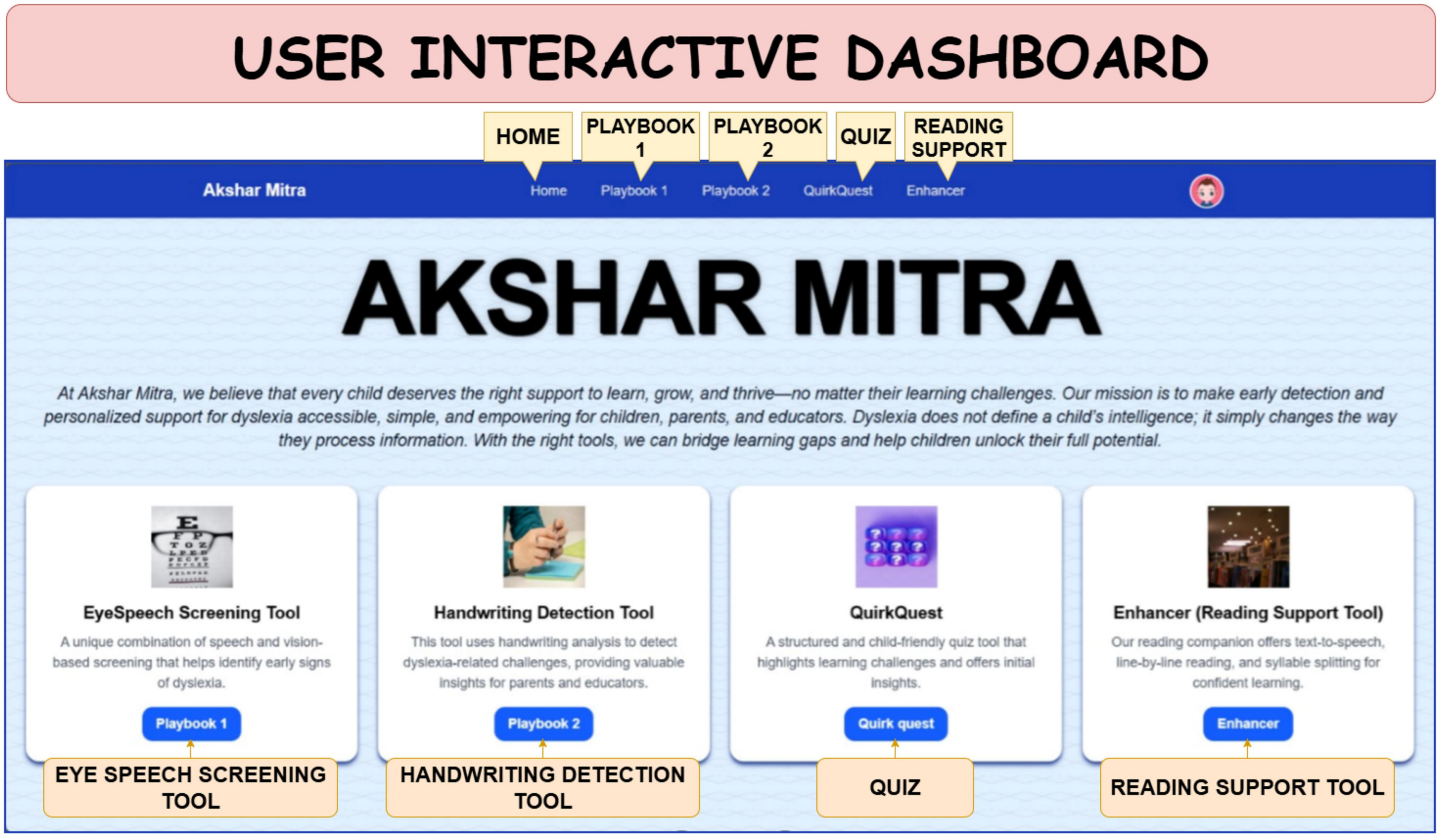
Dashboard of Akshar Mitra illustrating its modules.

Beyond detection, Akshar Mitra offers a dyslexia-friendly *Reading Companion* that incorporates evidence-based practices, large fonts, extra spacing, syllable-level breakdowns, and synchronized audio playback, to reduce cognitive load and enhance reading engagement. This study seeks to make early screening and intervention more accessible by designing a scalable, affordable, and child-focused solution, with special attention to reaching communities that face barriers due to limited resources or social marginalization. The User Interactive Dashboard of Akshar Mitra is illustrated in Figure 1, providing a high-level view of its components and workflow.

To guide the reader through this work, Section 2 reviews the state of the art in dyslexia detection and highlights key advances in multimodal approaches. Section 3 then introduces our proposed methodology, which integrates eye-tracking, speech recognition, and OCR-based analysis. Section 4 reports the experimental validation and results, while Section 5 concludes with key insights and future research directions.

## 2 State of the art

The traditional paradigm for the early identification of dyslexia, often reliant on psychometric evaluations and qualitative teacher observations, is undergoing a significant evolution driven by computational intelligence (11, 12). Recent scholarly work highlights a pivotal shift towards data-driven methodologies that offer the potential for more timely and objective screening. Specifically, the application of artificial intelligence (AI) and machine learning (ML) algorithms is enabling the analysis of complex, heterogeneous data to identify subtle neurodevelopmental markers indicative of dyslexia.

To systematically investigate the impact of this technology on dyslexia assessment, this review is structured around three pivotal areas of innovation. The analysis begins by examining modalities that capture fundamental processes impaired in dyslexia: reading and language. We first explore the use of Eye-Tracking for Dyslexia Identification, focusing on how quantitative analysis of saccades, fixations, and regressions provides objective biomarkers of reading difficulties (13, 14). Subsequently, the review investigates the role of Using Speech-to-Text in Dyslexia Diagnosis, delving into how automated analysis of spoken language can reveal underlying phonological deficits.

The scope then broadens to include another critical output skill: writing. This is covered under OCR and AI-Based Handwriting Analysis, which assesses how computational techniques can objectively evaluate graphomotor control and orthographic patterns from written samples. Finally, recognizing that a singular approach may be insufficient, the review culminates with an analysis of Multi-Modal Integration. This section critically evaluates advanced frameworks that combine data from two or more of the aforementioned modalities to enhance diagnostic accuracy and create a more holistic understanding of an individual’s neurodevelopmental profile.

### 2.1 Using eye-tracking for dyslexia identification

The application of eye tracking technology to examine the visual attention and behavior of children offers a timely, objective, and non-invasive way to study developmental disorders such as dyslexia. This technology precisely monitors the eyeballs by capturing and monitoring the eye movements through sophisticated cameras. Key eye tracking metrics include fixation counts, processing speed, saccades, scan path, dwell time and gaze duration (15). These measures provide important insights that can effectively differentiate between typical and dyslexic reading behaviors. Coupling machine learning with eye tracking data, they’ve demonstrated increased classification accuracy and promising possibility for scalable, early dyslexia screening in classrooms. Eye-tracking has also deepened our insight into the psychology of dyslexia by accurately recording eye fixations. Other ML models, including SVMs and CNNs, have also been deployed with great success on eye movement data to predict dyslexics (16, 17). Akshar-Mitra, a proposed solution, incorporates eye-tracking as a fundamental detection feature, likely utilizing it to monitor a child’s eye movements during reading tasks to identify patterns indicative of dyslexia (9).

### 2.2 Using speech-to-text in dyslexia diagnosis

Dyslexia, for instance, is associated with phonological processing deficits-a key factor that can be nicely tested through deep speech analysis. For example, AI-enabled speech recognition might evaluate a child’s oral language and detect particular problems with phonological or pronunciation awareness (18, 19). One new diagnostic direction here is to employ spectrogram analysis with CNNs. In this method, children’s raw audio waveforms are converted into spectrogram images which serve as input for CNN architectures. These models have demonstrated superior accuracy in identifying dyslexic individuals compared to traditional diagnostic methods. It shows up in every culture and language, and has similar prevalence in polyglot students as monolingual pupils. However, differentiating “normal” multi-lingual speech delays from an underlying learning disability is challenging and can result in late, over- or under- identification. Bilingual or native language screening is the key to fighting this, assessing such underlying constructs as phonological skills, rapid naming and working memory. While state-of-the-art AI offers the promise of accessing multilingual speech data, this area is also fraught with pitfalls, including language biases and the potential for underrepresented languages to be misrepresented in training data. Akshar-Mitra addresses this using speech recognition, specifically leveraging the OpenAI-Whisper API, to analyze spoken language and identify phonological problems. The system’s upcoming updates will also emphasize multilingual and local language support, which is critical for the intended user base.

### 2.3 OCR and AI-based handwriting analysis

Handwriting analysis using AI has emerged as a viable tool for the early detection of dyslexia and dysgraphia (20). The technology can identify several indicators, including misspelling, poor letter formation, and writing disorder. The AI models can identify motor difficulties through writing speed, pressure, and pen movement analysis as well as visual analysis of inconsistencies like irregular spacing and inconsistent letter size, common in people with these conditions. Moreover, AI can read cursive handwriting and digitize it, enable detection of common errors such as misspelling and letter reversal, and even determine underlying cognitive issues by reading grammar and vocabulary. Handwriting has consistently demonstrated unique and subtle features in comparison to the handwriting of dyslexic persons, including letter reversal and motor coordination issues that are amazingly revealing in describing neurological processing. One of the most crucial challenges with present-day AI handwriting analysis models is their transparency, which could make it difficult for teachers and clinisians to understand how specific handwriting features are driving a diagnosis.

However, one of the recurring issues still remains the paucity of diverse handwriting samples of children, which form the crux for effectively training robust AI models, particularly for varying populations.

Akshar-Mitra employs OCR handwriting recognition, with EasyOCR API, to analyze a child’s hand script for specific problems regarding written language processing (21, 26). An integrated multi-modal detection platform bringing together eye-tracking, speech recognition, and handwriting analysis offers a far more complete and perhaps even more accurate diagnostic profile than single-modality methods. This blending encompasses the many-splendored nature of dyslexia symptoms and minimizes dependence on any one, possibly erroneous, data source.

As algorithms for the dyslexia detection become increasingly sophisticated, their applicability in the real world and their effect in regards of fairness are severely constrained by the size, calibration, and diversity of training data. This underlines a key gap for research: that there is a need for large representative datasets that are culturally and linguistically diverse to harness the maximum potential of AI for the diagnosis of dyslexia internationally, especially in multilingual environments such as India. For AI-based dyslexia screening tools to achieve broad confidence and usage by educators, parents, and clinicians, high accuracy is not enough. These challenges underscore a critical research direction: the development of systems that are not only inclusive but also capable of leveraging multimodal indicators of dyslexia to enhance reliability and generalizability.

## 3 Materials and methods

**Figure 2:**
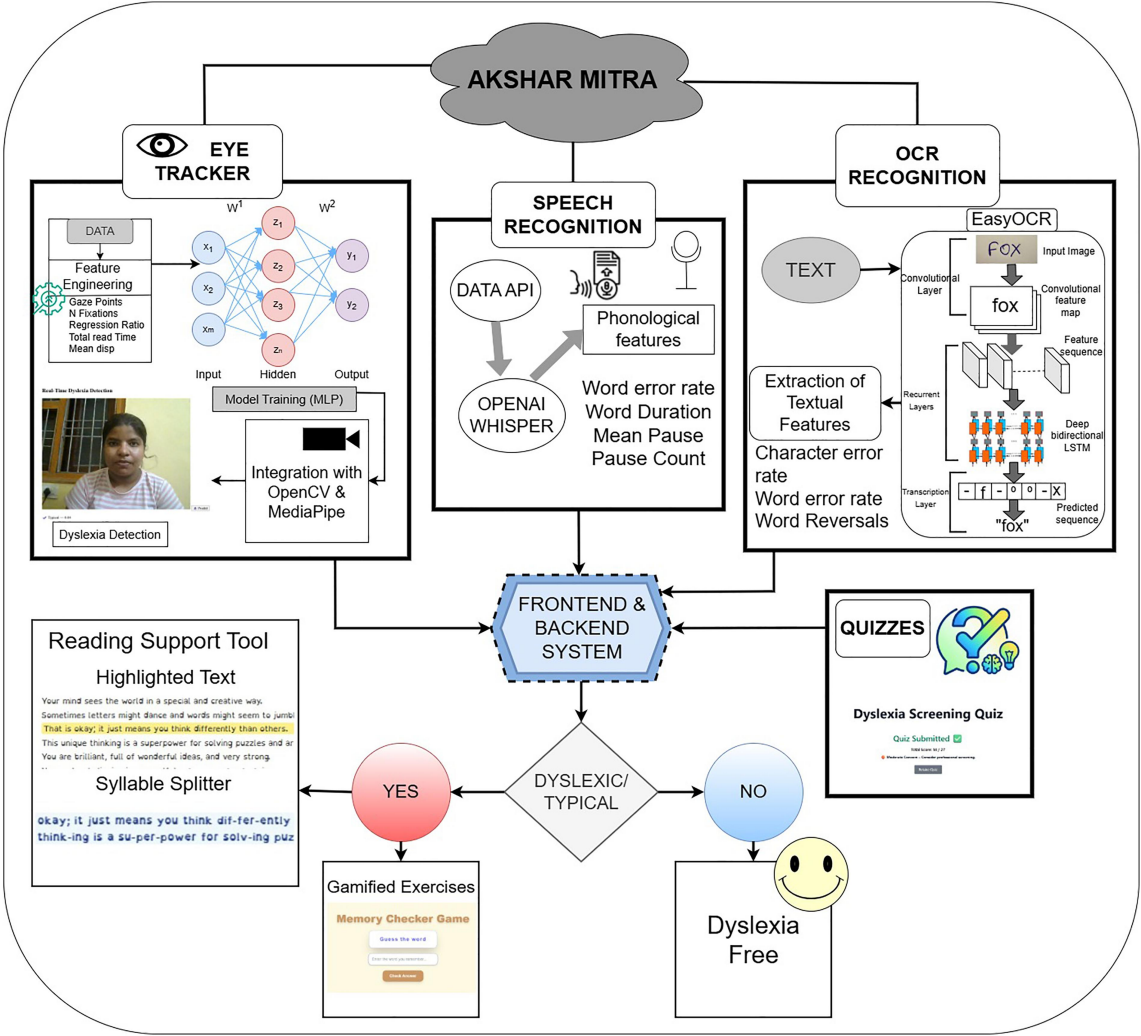
Workflow of Multimodal Framework (MMF) consisting of various features.

This work introduces Akshar Mitra (Framework for Reading, Interpretable Eye-tracking, Dyslexia Screening), a system developed upon the Multimodal Framework (MMF) and is given in Figure 2. The architecture is composed of six interoperable modules, engineered under a unified design to facilitate future multimodal fusion for enhanced screening accuracy.

The system processes three primary data streams in parallel: (1) video for eye-gaze tracking, (2) audio for speech signal analysis, and (3) handwriting samples from digital ink . These core inputs are supplemented by a behavioral assessment module and a reading assistance tool. A dedicated processing pipeline with three parallel encoders performs feature extraction, generating a compact set of 4-6 interpretable features for each data stream. To ensure system-wide compatibility and support real-time inference, all features are standardized into a common schema. This modular architecture is explicitly designed to serve as a foundation for a future unified dyslexia classification model, the details of which are elaborated in subsequent sections.

### 3.1 Eye-tracking-based fixation analysis

Eye-tracking has been established as a non-invasive and objective method for investigating the neurocognitive processes underlying reading. By analyzing oculomotor metrics during text comprehension, it is possible to identify distinct patterns associated with developmental dyslexia. Empirical evidence consistently demonstrates that individuals with dyslexia exhibit longer fixation durations, a higher frequency of regressive saccades (backward eye movements), and a lower overall reading speed when compared to typical readers. These quantitative metrics serve as robust biomarkers for distinguishing between dyslexic and neurotypical reading behaviors. The integration of these oculomotor features with machine learning algorithms has yielded high-performance classification models, with recent systems achieving detection accuracies of approximately 92% using eye-tracking data alone. This convergence of objective oculomotor analysis and computational modeling provides a powerful paradigm for developing rapid screening tools. Building upon this foundation, this paper introduces a novel Eye-Tracking-Based Dyslexia Screening Pipeline given as Algorithm 1.

Algorithm 1 **Eye-Tracking-Based Dyslexia Screening Pipeline**

**Require:** Folder of fixation CSVs, webcam gaze stream

**Ensure:** Trained classifier *M* and real-time dyslexia label $\hat{y}$

 **Offline Training:**

1:**function** TrainClassifier(input_dir)

2:  F←∅        ▹ initialize empty feature set

3:  **for all** file in ListFiles(input_dir, “*_fixations.csv”) **do**

4:    *metrics* ← ExtractFeaturesFromFile (*file*)

5:    **if** metrics≠None **then**

6:     Append(*F*, metrics)

7:    **end** **if**

8:  **end for**

9:  D←DataFrame(F).DropMissing()

10:  Split *D* into $\left(X_{\mathrm{train}},y_{\mathrm{train}}\right)$ and $\left(X_{\mathrm{test}},y_{\mathrm{test}}\right)$

11:  Normalize features in $X_{\mathrm{train}},X_{\mathrm{test}}$

12:  Train MLP classifier *M* on $\left(X_{\mathrm{train}},y_{\mathrm{train}}\right)$

13:  Evaluate *M* on $\left(X_{\mathrm{test}},y_{\mathrm{test}}\right)$ using accuracy, $F_{1}$

14:  **return** trained model *M* and normalizer

15: **end function**

16: **function** ExtractFeaturesFromFile(file_path)

17:  Load CSV into df; if empty, **return** None

18:  Convert duration_ms, disp_x, disp_y to numeric

19:  Compute:

n_fix←|df|

mean_fix←mean(duration_ms)

regressionj_ratio←mean(disp_x<0)

lines_switches←df.aoi_line.nunique()

20:  total_read_time←max(end_ms)−min(start_ms)

21:  **return** dictionary of metrics

22: **end function**

 **Online Inference:**

23: **function** PredictFromWebcam(gaze_points, M, normalizer)

24:  **if** |gaze_points|<5 **then**

25:   **return** error “insufficient data”

26:  **end if**

27:  H←deque(maxlen=100)

28:  **for all** pt in gaze_points **do**

29:    Append(H, pt)

30:  **end** **for**

31:  feats←ExtractGazeFeatures(H)

32:  **if** feats=None **then**

33:    **return** error “feature extraction failed”

34:  **end if**

35:  $feats^{\prime }\leftarrow \mathrm{normalizer}.\mathrm{transform}(feats)$

36:  $\hat{y},p\leftarrow M.\mathrm{predict}\_\mathrm{proba}(feats^{\prime })$

37:  **return** label $\hat{y}$ with confidence p

38: **end function**

The proposed pipeline operationalizes these research principles into a practical and efficient system. It is specifically engineered to utilize a minimalist set of the most salient oculomotor biomarkers, ensuring computational efficiency and robustness for real-time application while maintaining high diagnostic accuracy.

#### 3.1.1 The ETDD70 dataset

This study utilizes the publicly available ETDD70 eye-tracking corpus for model development and validation. The dataset comprises recordings from 70 Czech schoolchildren (aged 9–10), evenly distributed between diagnosed dyslexic and typical reader groups (22). The experimental protocol required participants to read three distinct passages: a syllable matrix, a narrative story, and a pseudo-text. Raw gaze data were processed into fixation and saccade events using the I2MC algorithm, which was selected for its robustness against noise commonly found in pediatric eye-tracking recordings, with a minimum fixation duration threshold of 40 ms (22).

While a comprehensive set of features can be derived, the logistical challenges of high-fidelity eye-tracking make such datasets relatively small, posing a significant risk of model overfitting due to inherent signal variability. Therefore, to ensure model generalizability and interpretability, in this work a stringent feature selection process is adopted, where four trial-level features are identified as having the highest discriminative power between the dyslexic and typical reader cohorts.

#### 3.1.2 Feature selection and justification

A feature selection process was conducted to identify a minimal yet highly discriminative set of metrics for dyslexia classification. From an initial pool of candidates that included fixation variance (*std_fix*) and spatial dispersion (*mean_disp_x*, *std_disp_x*), the final feature set was refined to four key metrics: Fixation Count (*n_fix*), Mean Fixation Duration (*mean_fix*), Regression Ratio (*regression_ratio*), and Total Reading Time (*total_read_time*). The selection was guided by three primary criteria: (i) strong empirical support from prior dyslexia research, (ii) high signal robustness across both controlled and unconstrained webcam-based conditions, and (iii) low redundancy as determined through feature ablation studies. Metrics such as spatial dispersion and line-switch frequency were ultimately discarded due to their high variance and poor generalizability, particularly under the variable lighting conditions inherent to webcam-based eye-tracking.

The selected four-feature set offers significant clinical and computational advantages. Each metric directly corresponds to a well-documented oculomotor characteristic of dyslexia, namely increased fixation frequency, prolonged gaze durations, a higher proportion of regressive saccades, and slower task completion. This minimalist design facilitates stable, real-time inference with minimal computational overhead, ensuring viability in resource-constrained environments like mobile devices or low-resolution video streams while maintaining high classification performance. The resulting feature set thus represents an optimal balance between diagnostic sensitivity, efficiency, and robustness to signal noise, rendering it highly suitable for scalable deployment in clinical and educational settings.

#### 3.1.3 Machine-learning classification

The classification of the standardized features is performed by a Multilayer Perceptron (MLP) . The network architecture comprises a single hidden layer with 64 neurons utilizing a Rectified Linear Unit (ReLU) activation function. The model was optimized using the Adam algorithm with a cross-entropy loss function. For training and evaluation, the dataset was partitioned via stratified sampling into an 80% training set and a 20% held-out validation set to maintain class balance. On the validation data, the classifier achieved an accuracy of approximately 92% and a high F1-score in distinguishing between dyslexic and typical readers. For deployment, the trained model weights and the feature standardization scaler are stored. The inference pipeline first transforms a new feature vector using the saved scaler and then inputs it into the MLP to generate a final dyslexia probability score.

#### 3.1.4 Integrated real-time eye-tracking and speech-based dyslexia screening system

The proposed system integrates a real-time eye-tracking module with a speech analysis component to enable multimodal dyslexia screening during controlled reading sessions. In this setup, a webcam-based gaze tracking application operates concurrently with an audio capture module while the participant reads a displayed sentence aloud.

A continuous video stream is processed on a per-frame basis, where facial landmarks are detected using the MediaPipe, FaceMesh framework to accurately estimate the eye regions. From each processed frame, an approximate gaze point is calculated by averaging predefined eye landmarks corresponding to the pupil and surrounding ocular features. These gaze coordinates are appended to a short-term buffer, typically containing the most recent 100 observations, thereby preserving the temporal evolution of eye movements throughout the reading task.

Once a sufficient number of samples are accumulated, or upon receiving a trigger signal (such as completion of the spoken sentence), the system computes four primary gaze-based features:

1. Number of fixations
2. Mean gaze velocity
3. Count of regressive (backward) saccades
4. Total duration of observation

These features are normalized and passed as input to a pretrained neural classification model, which outputs a probability score indicating the likelihood of dyslexia. The real-time inference result is dynamically overlaid onto the video feed, displaying indicative labels such as “Typical” or “Dyslexic” along with their corresponding confidence values. All feature vectors, predictions, and timestamps are securely logged for subsequent offline analysis. The end-to-end gaze tracking pipeline is optimized for low-latency operation, ensuring interactive performance during live sessions.

Simultaneously, the speech analysis module records and transcribes the participant’s spoken output using an Automatic Speech Recognition (ASR) framework. The transcribed text is compared against a predefined reference passage to derive several linguistic and temporal indicators, including:

1. Word Error Rate (WER)
2. Reading speed (words per minute)
3. Frequency and total duration of pauses during reading

Upon completion of the session, the gaze-derived and speech-derived features are jointly analyzed to generate a final dyslexia risk score. This fusion provides a comprehensive assessment by integrating visual attention dynamics with speech fluency and accuracy metrics.

All session data—including raw gaze coordinates, processed features, transcribed speech, and model outputs—are securely stored in an ethically managed institutional database, thereby enhancing the dataset for supervised training and evaluation of future multimodal dyslexia detection models.

This integrated implementation demonstrates the feasibility of real-time, camera-based dyslexia screening that is both portable and non-invasive. By combining gaze movement patterns with speech characteristics, the system provides a reliable framework for early dyslexia risk detection in real-world educational and clinical environments. The detailed operational design and processing pipeline of the speech analysis module are discussed in the following section.

### 3.2 Speech fluency and timing analysis

The current prototype for speech-based dyslexia screening utilizes an Automatic Speech Recognition (ASR) engine (OpenAI’s Whisper) to process recordings of a child reading a fixed text. From the resulting transcription, quantitative features such as Word Error Rate (WER), pause statistics, and speaking rate in Words Per Minute (WPM) are computed against the known ground-truth passage. Dyslexia risk is subsequently assessed using a preliminary, heuristic-based algorithm. While this approach has provided initial validation, its performance is fundamentally constrained by a dependency on these heuristics and the absence of a large, curated dataset for training a robust classification model.The stepwise implementation of this process is formalized in the following Algorithm 2

Algorithm 2 **Speech-Based Dyslexia Feature Extraction and Prediction**

**Require:** Reference transcript, Actual transcript, Word timing list

**Ensure:** Extracted features, Dyslexia prediction

1: Compute **WER** = Word Error Rate between reference and transcript

2: Initialize pauses list

3: **for** *i* = 1 to length of words **do**

4: pause = words[*i*].start − words[*i* − 1].end

5: **if** pause >0 **then**

6:  Append pause to pauses

7: **end if**

8: **end for**

9: Compute word durations = end − start for each word

10: Compute total_time = last word end − first word start

11: Compute speech_time = sum of all word durations

12: Calculate features:

- Mean word duration
- Mean pause
- Pause count where pause >0.2 sec
- Words per minute (WPM) = #words/total_time × 60
- Articulation rate = #words/speech_time

13: Initialize score = 0

14: **if** WER ≥0.15 **then**

15:  score ← score + 1

16: **end if**

17: **if** pause_count ≥5 **then**

18:  score ← score + 1

19: **end if**

20: **if** WPM ≤ 80 **then**

21:  score ← score + 1

22: **end if**

23: **if** score ≥ 2 **then**

24:  **return** “Likely Dyslexia”

25: **else**

26:  **return** “Typical”

27: **end if**

To overcome these limitations, future work is focused on transitioning from the current rule-based system to a data-driven framework. Key objectives include: developing robust methods for precise, forced alignment of the speech signal with the reference text; expanding the feature set to include more informative acoustic and prosodic markers identified in child speech and dyslexia research; and establishing a systematic data collection pipeline, complete with automated report generation, to build a dedicated training corpus.

#### 3.2.1 Child-adaptive ASR and transcription

Children’s speech differs acoustically and linguistically from adults’. Thus, using an ASR model adapted to children significantly improves transcription accuracy.

Recent work shows that fine-tuning Whisper on child speech corpora yields lower WER. Jain et al., report that Whisper fine-tuned on 55h of child speech reduces WER on reading tasks.

Fine-tuning Whisper with child-specific corpora (CUCHILD) is advisable. Subsequent adaptation can be performed using task-specific labeled data from the target demographic.

#### 3.2.2 Key features for dyslexia screening

The rule-based speech model incorporates a foundational set of features, primarily targeting prosodic and temporal aspects of reading. The feature space is extended to include reading rate, quantified as words per minute (WPM) or syllables per second. Children at risk of dyslexia often exhibit slower reading speeds accompanied by frequent pauses; this is captured by analyzing aligned timestamps together with pause statistics (e.g., number and duration of pauses longer than 200 ms).

Pronunciation accuracy is modeled through phoneme-level edit distances derived from forced alignment, with the Goodness of Pronunciation (GOP) scoring framework applied to detect mispronunciations. An increased phoneme-error rate is used as an indicator of decoding difficulties.

Rhythmic irregularities are characterized by extracting measures such as pause duration variability and articulation rate. These features provide insight into disrupted prosody, which is a recognized marker of dyslexic speech patterns. While the current focus remains on timing- and pronunciation-based variables, the framework is designed to be extensible. Future extensions may incorporate higher-level prosodic markers such as pitch variation, stress distribution, and phrase boundary detection. Incorporating these variables has demonstrated improvements in both classification performance and interpretability.

### 3.3 Handwriting OCR and symbolic error evaluation

This module presents an automated pipeline to detect dyslexia-associated writing difficulties using a scanned or photographed image of a handwritten sentence.The algorithm is given as Algorithm 3

Algorithm 3 **OCR-Based Dyslexia Risk Assessment System**

**Require:** Image of handwritten text, Expected sentence

**Ensure:** Dyslexia risk level, performance metrics

1: Initialize EasyOCR reader

2: Define reversal pairs: {(“b”,“d”), (“d”,“b”), (“p”,“q”), (“q”,“p”)}

3: Normalize expected sentence (Unicode-aware lowercase)

4: Receive uploaded image and expected sentence from user

5: **if** missing input or sentence exceeds 500 words **then**

6:  **return** Error message

7: **end if**

8: Save uploaded image temporarily

9: Apply OCR to extract text → actual text

10: **if** OCR fails to recognize any text **then**

11:  **return** Error message

12: **end if**

13: Normalize actual OCR output

14: Initialize counters: substitutions, insertions, deletions

15: Compare expected vs. actual using SequenceMatcher

16: **for all** edit operations (tag, i1, i2, j1, j2) **do**

17:  **if** tag is “equal” **then**

18:    Mark characters as correct

19:  **else**

20:    Mark characters as incorrect

21:    Update corresponding counters

22:   **end if**

23: **end for**

24: Calculate Character Error Rate (CER)

25: Calculate Word Error Rate (WER)

26: Count letter reversals using defined pairs

27: Compute dyslexia risk score based on:

- CER and WER
- Substitutions, Insertions, Deletions
- Letter reversals

28: Classify risk as:

- **High:** Recommend screening
- **Moderate:** Monitor further
- **Low:** No significant indicators

29: **return** JSON output with all metrics and risk level

#### 3.3.1 Image processing and OCR

The pipeline uses Optical Character Recognition (OCR) to extract printed text and performs fine-grained comparison against the expected text to detect orthographic inconsistencies. The system is lightweight, deployable in clinical or classroom environments, and capable of real-time analysis and report generation. Users are prompted to write a fixed seed sentence (e.g., “The quick brown fox jumps over the lazy dog”) and upload an image of their handwriting via a web interface. The image is processed using *EasyOCR*, a convolutional neural network–based OCR system pre-trained on English scripts. EasyOCR returns a linearly ordered character sequence (actual) from the image without needing complex preprocessing, bounding box filtering, or layout correction, making it robust to the variability present in children’s handwriting.

#### 3.3.2 Normalization and sequence alignment

Both the reference sentence (expected) and the OCR output are normalized using Unicode normalization (NFKC) and lowercased to reduce orthographic variance. The resulting strings are compared using a dynamic sequence alignment algorithm (*difflib.SequenceMatcher*) that generates character-level edit operations: substitutions, insertions, and deletions. The goal is to identify discrepancies in symbol representation attributed to handwriting errors, OCR artifacts, or spelling problems.

Two primary distance metrics are computed to quantify the deviation from the reference sentence: the Character Error Rate (CER) and the Word Error Rate (WER). The CER is defined as:

$$\text{CER}=\frac{\text{substitutions}+\text{insertions}+\text{deletions}}{\text{number of characters in reference}}$$

This metric captures character-level deviations. The WER, on the other hand, is calculated by tokenizing both the reference and hypothesis texts and computing the word-level edit distance between them. These metrics provide a quantitative assessment of transcription fidelity and are utilized as core features in the final dyslexia screening decision.

#### 3.3.3 Symbol reversal detection

An additional dyslexia-specific feature is the **letter reversal count**, defined as the number of character pairs that match common visual confusions such as:

(b↔d),(p↔q),(q↔p)

Such reversals are hallmark indicators of visual-spatial confusion and dysgraphia in early dyslexic children. They are detected via a character-wise scan of the aligned strings and contribute heavily to the screening score.

#### 3.3.4 Rule-based risk scoring

Based on literature-derived thresholds and empirical tuning, a weighted scoring model is implemented. For each sample, the following features are computed: substitution count, insertion count, deletion count, reversal count, character error rate (CER), and word error rate (WER). Each feature contributes one or two points depending on the severity of deviation.

The total score is then mapped to one of three discrete risk classes. A score greater than or equal to 7 (23) indicates high risk, suggesting that immediate and detailed screening is recommended. Scores between 4 and 6 correspond to a moderate risk category, where monitoring for patterns and retesting is advised. A score below 4 reflects a low risk assessment, implying that no significant dyslexia indicators were found.

This rule-based structure is designed for interpretability and can be updated with weights learned via machine learning once labeled handwriting data is available.

#### 3.3.5 Report generation and user feedback

The system utilizes the xhtml2pdf library to convert a Jinja2-based HTML template into a downloadable PDF diagnostic report. This report includes the uploaded handwritten image, the OCR result alongside the expected sentence, and a visual comparison highlighting symbol matches and mismatches. It also contains all extracted metrics, such as character error rate (CER), word error rate (WER), and reversal count, along with the final risk classification message. These reports can be archived or shared with clinicians or educators for deeper follow-up. Additionally, this functionality facilitates anonymized data collection to support future supervised learning tasks.

### 3.4 Behavioral quiz-based screening

To supplement objective modalities, a digital questionnaire targeting behavioral indicators commonly associated with dyslexia was developed. The quiz consists of 9 clinically inspired multiple-choice items (e.g., reversal confusion, difficulty rhyming, inconsistent spelling) derived from established dyslexia symptom checklists and is given in Appendix. Each question is scored on a 4-point Likert scale (0–3). The total score ranges from 0 to 27, and risk levels are categorized using fixed thresholds (Table 1).

**Table 1:** Risk interpretation based on dyslexia screening score.

Score range	Interpretation
0–6	Unlikely dyslexia - No significant signs.
7–13	Mild signs - Monitor and support.
14–20	Moderate concern - Consider professional screening.
21+	High concern - Seek professional evaluation.

The quiz is implemented as a React-based web component, allowing for real-time scoring, progress navigation, and retakes. The responses are stored locally for privacy, with potential future integration into the unified backend for multimodal fusion.

Although subjective, the quiz serves as a lightweight behavioral filter to flag potential concerns when objective modules (e.g., eye-tracking) may be unavailable. It is especially useful in remote or early-stage screening, guiding decisions on whether to proceed with deeper testing.

### 3.5 Support-oriented interventions

While dyslexia is a lifelong neurological condition with no definitive cure, early detection enables timely support to improve reading outcomes and confidence. To bridge the gap between diagnosis and intervention, two supportive modules integrated into this broader framework was proposed:

(a) **Reading Support System:** To assist children after screening, a lightweight *Reading Support System* was developed that emphasizes syllable-level decoding and pacing adjustments. The system is implemented as a Flask-based web service that interfaces with a React or static front-end, enabling seamless integration into classroom or home environments.

#### 3.5.1 Architecture and workflow

**Figure 3:**
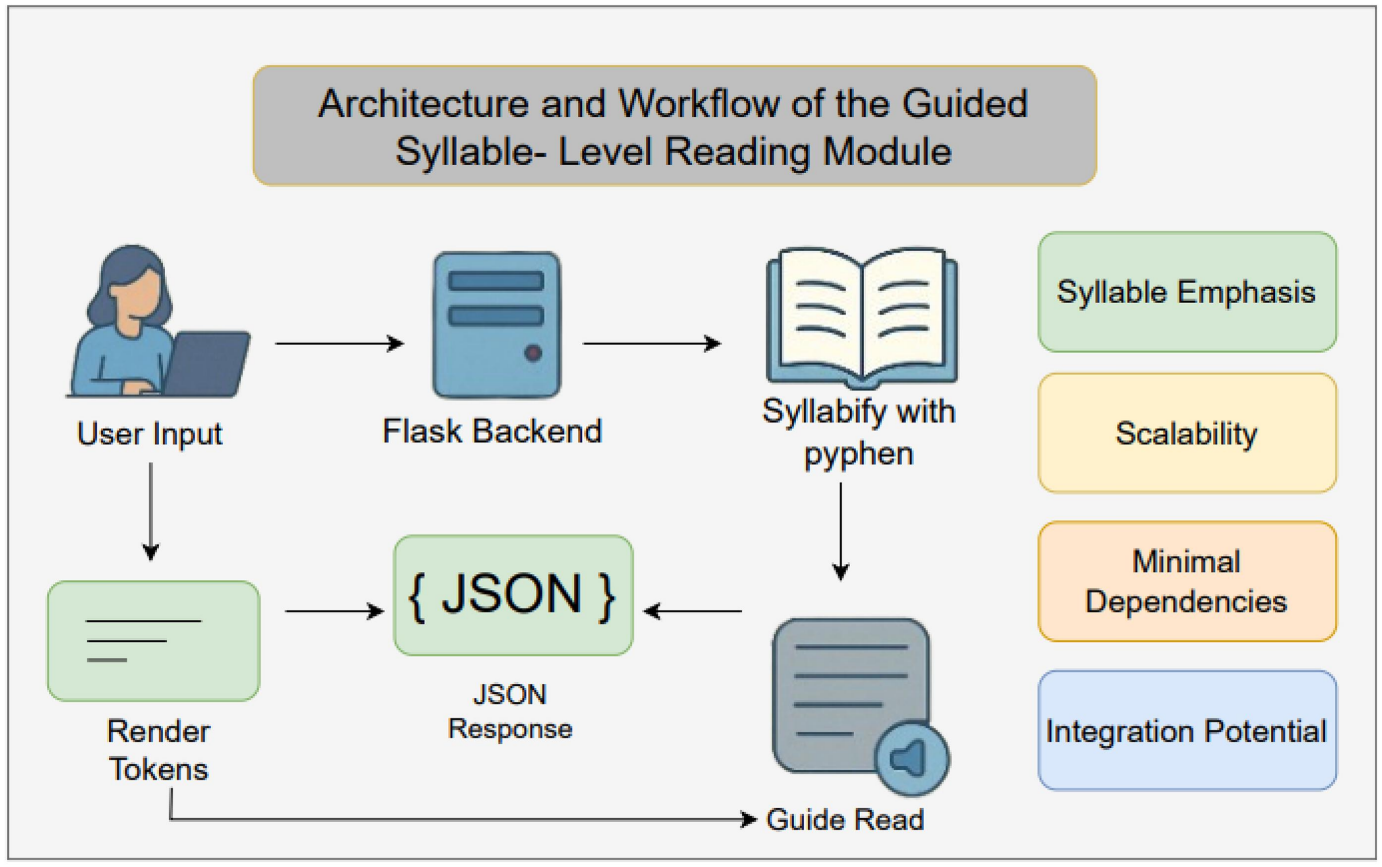
Workflow of the guided syllable-level reading module.

Workflow of the guided syllable-level reading module is given in Figure 3.

#### 3.5.2 Key features

The proposed module incorporates several important characteristics that ensure both usability and efficiency. Table 2 highlights the key features of the syllable-based reading module.

**Table 2:** Key features of the syllable-based reading module.

Feature	Description
Syllable emphasis	Reinforces phonological decoding by isolating syllables, reducing cognitive load for struggling readers.
Scalability	Handles passages up to 5,000 words, supporting full-length texts without performance issues.
Minimal dependencies	Requires only Flask, flask_cors, and pyphen, enabling easy deployment even on low-resource systems.

#### 3.5.3 Integration potential

Although currently a standalone tool, the Reading Support System is designed to interoperate with the other modules in this framework. For example, gaze coordinates from real-time eye tracking (Section 3.1) can trigger automatic syllable advancement, while back-end logs of syllable dwell times could feed into future machine learning models to personalize pacing.

#### 3.5.4 User experience

Clinicians or educators can upload or paste any instructional text; the system instantaneously returns a syllabified version. Children follow on-screen highlights or listen to synchronized text-to-speech (TTS) in future iterations, promoting fluency and confidence.

This intervention bridges the gap between early detection and targeted support, providing an immediately deployable tool to reinforce phonological skills in real time.

(b) **Gamified Learning Interface:** A game-based environment is being developed in which children engage in letter-sound matching, sequencing tasks, and rhyming puzzles. The gamified design promotes motivation, reduces stigma, and reinforces skills in an adaptive, low-pressure setting.

These modules are not diagnostic but therapeutic, intended to complement the screening tools. Future iterations will integrate user performance into the unified data stream, enabling personalized interventions and progress monitoring over time.

## 4 Experimental validation and results

The initial testing has demonstrated promising result with controlled dataset as outcomes. The system was able to flag potential dyslexic traits in children, through the interactive assessment. The eye tracker, Speech to Text, OCR and AI-based Handwriting Analysis are the assessments used in the application. The user feedback indicate that the interface was intuitive and helpful particularly for non-specialist users such as parents or primary school teachers. The application also meets the requirement in the assisting of the dyslexic children.While the system is not a diagnostic tool, it effectively supports early screening objectives.

The Akshar Mitra home page presents all six modules-Eye Tracking, Speech Fluency, Handwriting OCR, Behavioral Quiz, Reading Companion and Gamified tools-as interactive cards on a single dashboard. Users can simply click a module’s card to navigate directly to its testing interface and begin assessment.

### 4.1 Performance analysis

**Figure 4:**
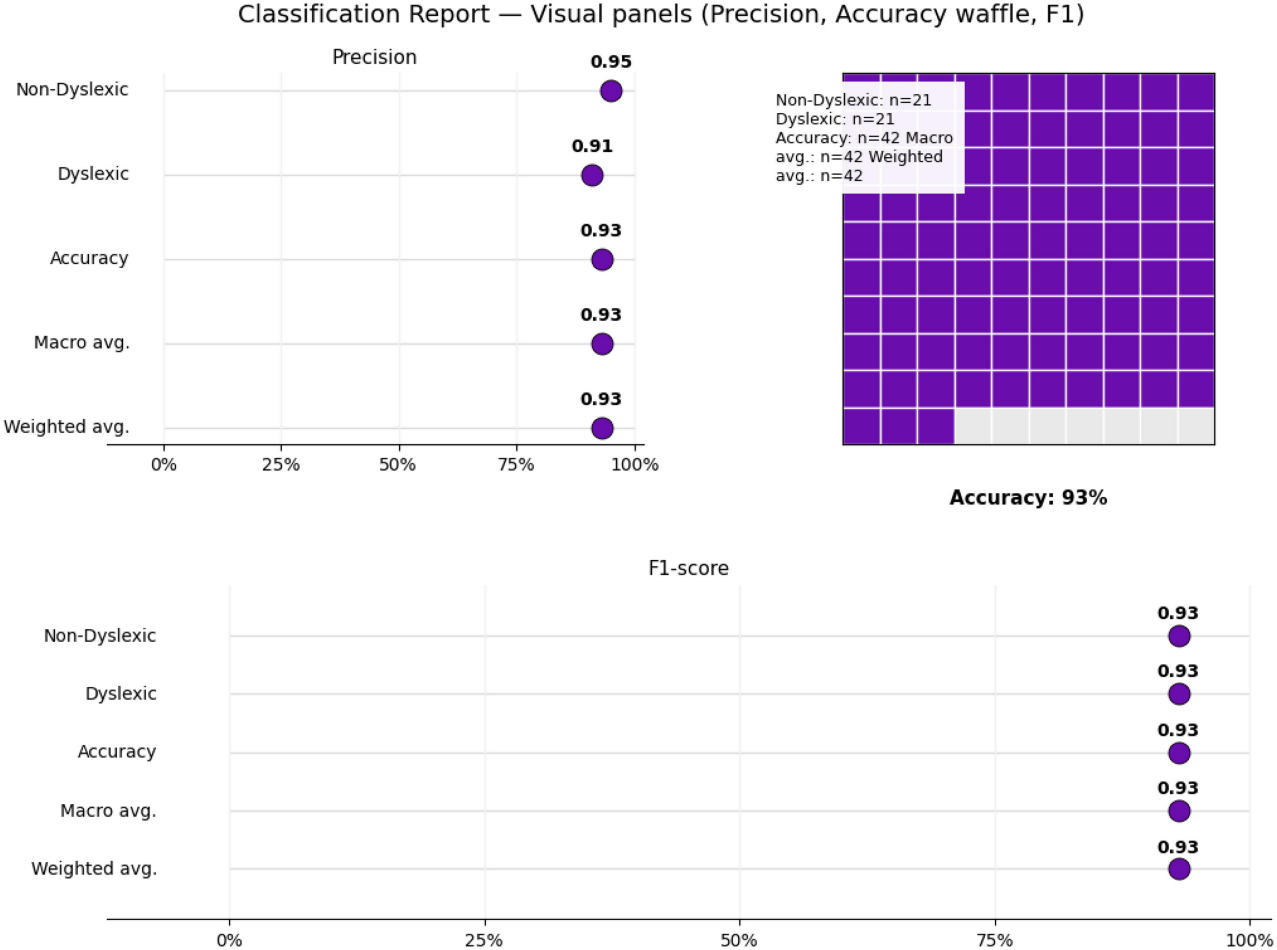
Classification report suggesting accuracy, recall, precision and f1-score along with the support.

**Figure 5:**
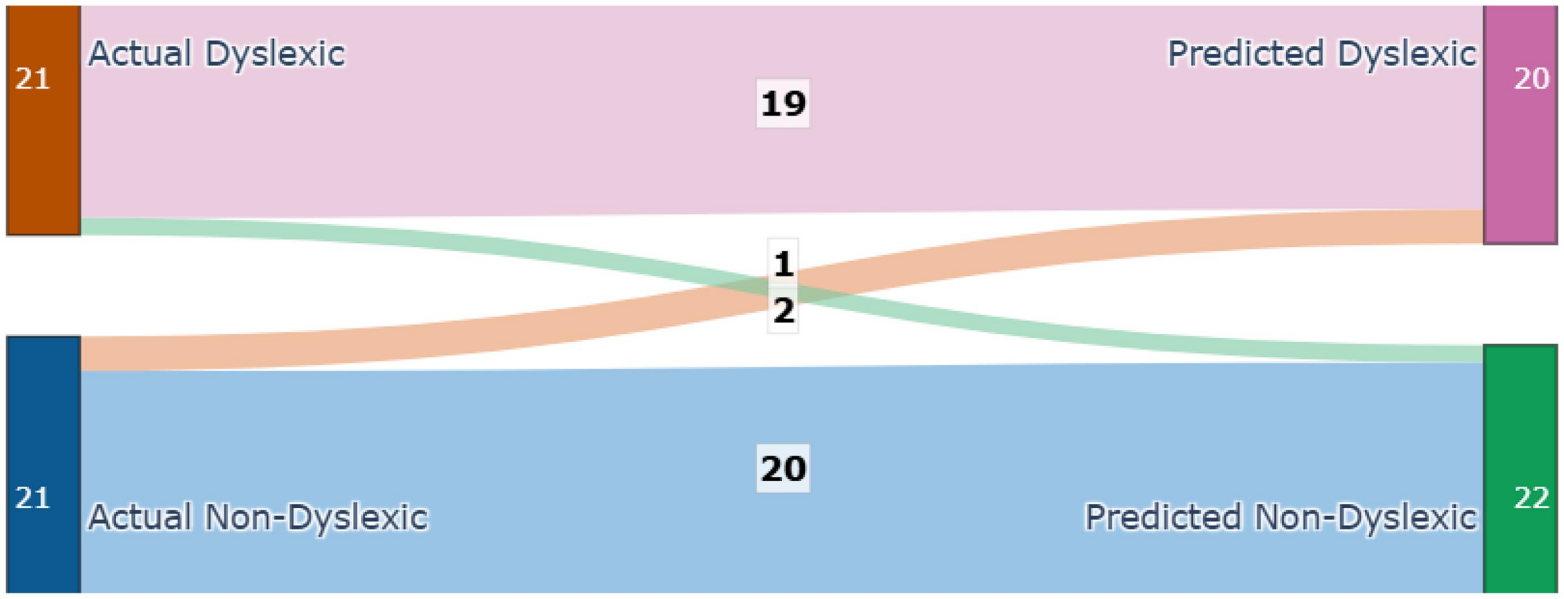
Confusion matrix for eye-tracker.

**Figure 6:**
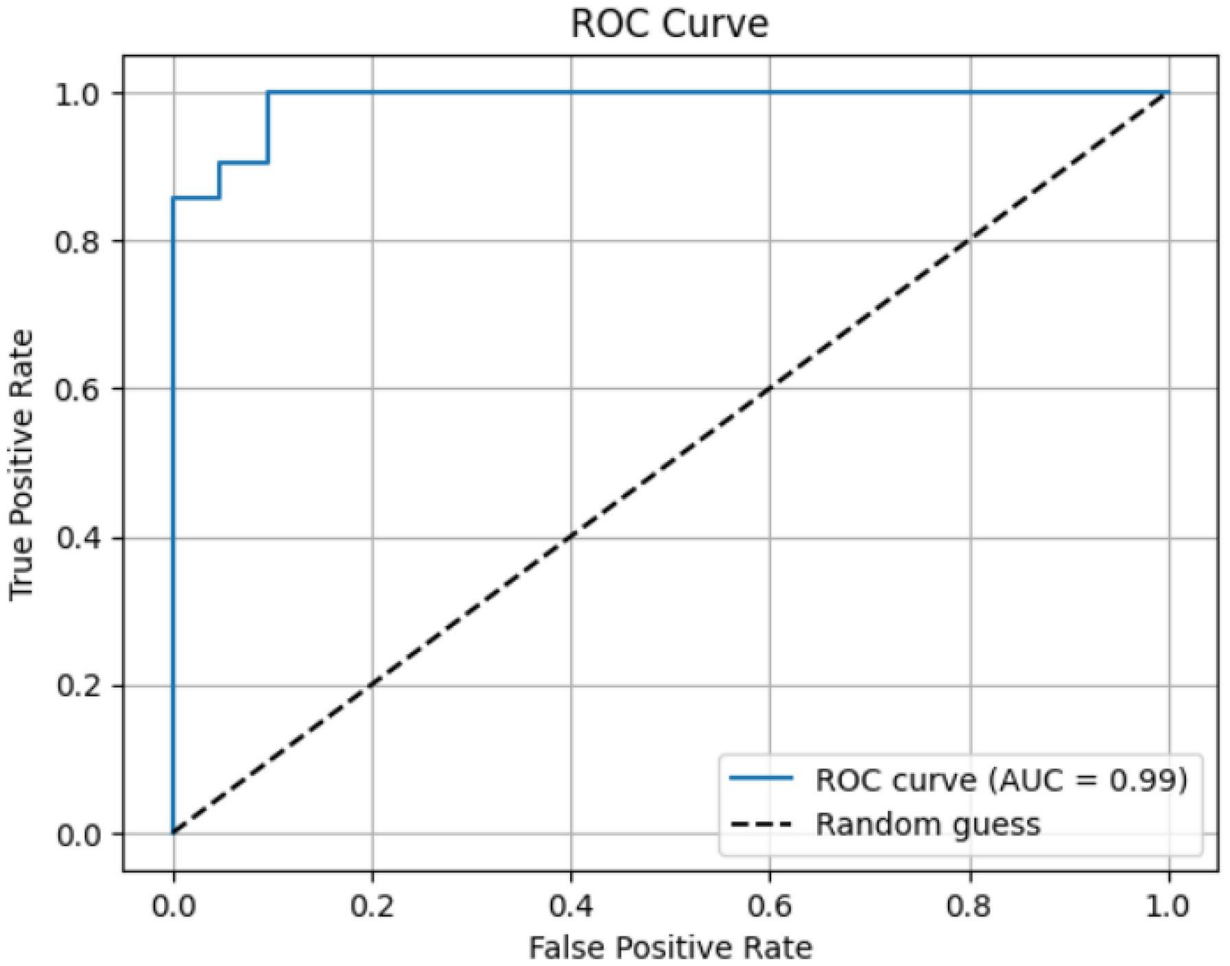
ROC curve.

Each module was evaluated on its respective test set using accuracy, precision, recall Figure 4, F_1_-score, confusion matrices Figure 5, and ROC analysis Figure 6. The eye-tracking classifier achieved an accuracy of 92.8%, and an F_1_-score of 0.93, its ROC curve yielded an AUC of 0.99, with the confusion matrix showing low false positive and negative rates. These results demonstrate that the eye-tracking classifier reliably distinguishes dyslexic from typical readers, with confusion matrices indicating a well-balanced distribution of classification errors across both classes.

#### 4.1.1 Eyes and speech analysis feature

**Figure 7:**
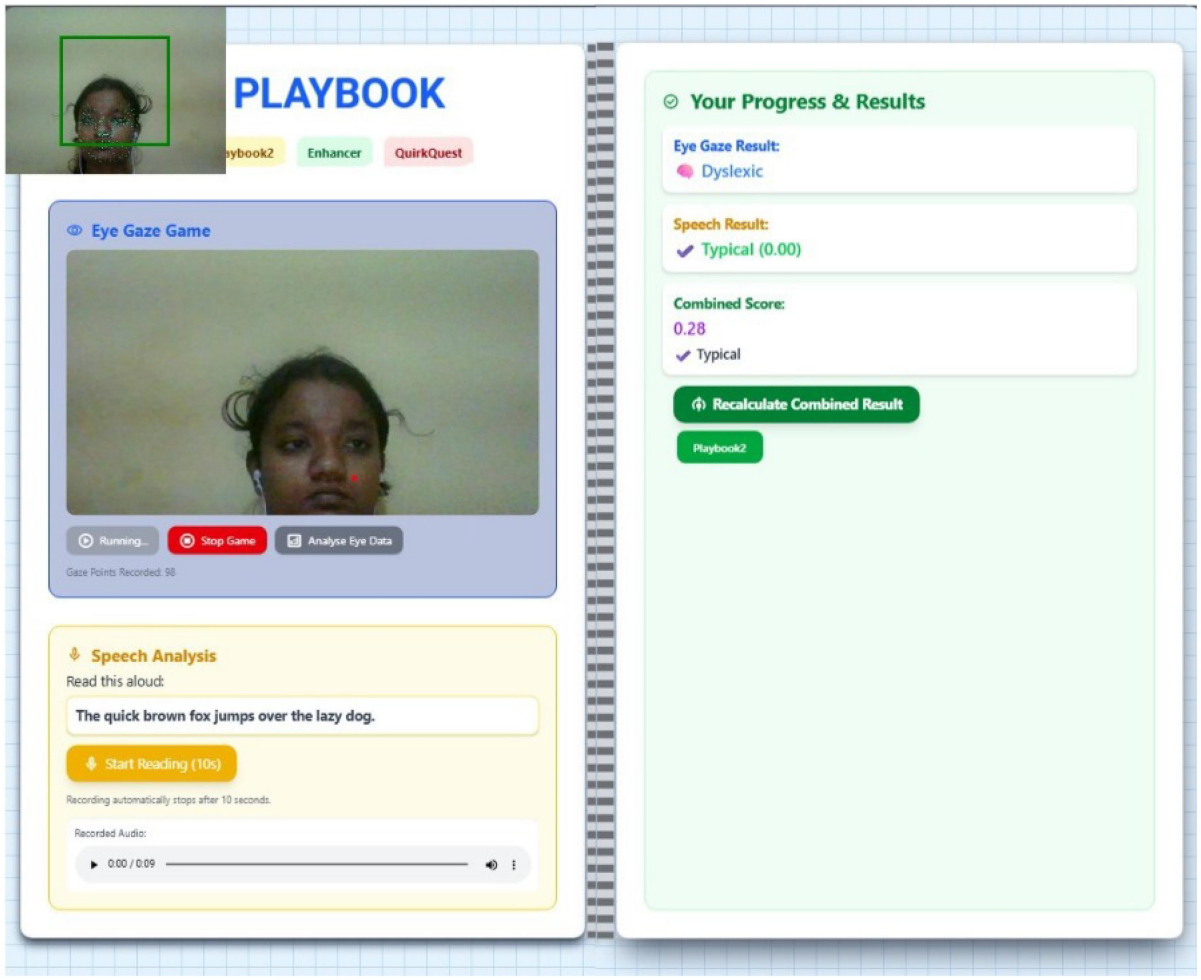
Eyes and speech analysis for typical and dyslexic cases.

This work describes a new multimodal system developed as a tool for dyslexia screening, which integrates two diagnostic modules-Eye-Gaze Analysis and Speech Analysis-under a unified digital framework Figure 7, 8. The user protocol is comprised of two sequential tasks: an eye-tracking session, lasting 10 s, that gathers and validates the required number of eye-landmark samples, followed by a speech task lasting 10 s in which the user reads a prescribed sentence. The two modalities are processed separate then the aggregated eye-gaze features are entered into a trained classifier that outputs an initial estimate, whilst the recorded audio is processed as input to estimate the Word Error Rate (WER) and provide an Automatic Speech Recognition (ASR) transcript. Here, the innovation in the system is the sophisticated combination of these outputs into a “Combined Score” that is rendered along with the individual outputs. By comparing quantitative indicators (gaze patterns, WER) with qualitative data (playback of the recorded audio, transcripts), this unified system provides a comprehensive, multifaceted interpretation of reading proficiency, thereby providing a more reliable and subtle screening output that could be achieved through unimodal analysis alone.

**Figure 8:**
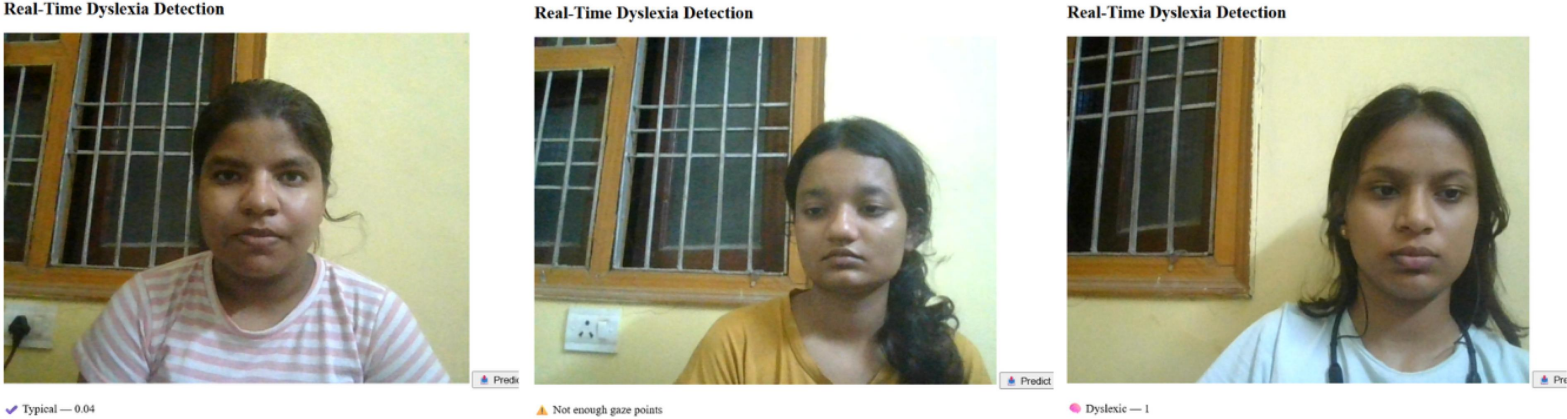
Eye tracker tested on various individuals showing different results.

#### 4.1.2 OCR-based dyslexia screening

**Figure 9:**
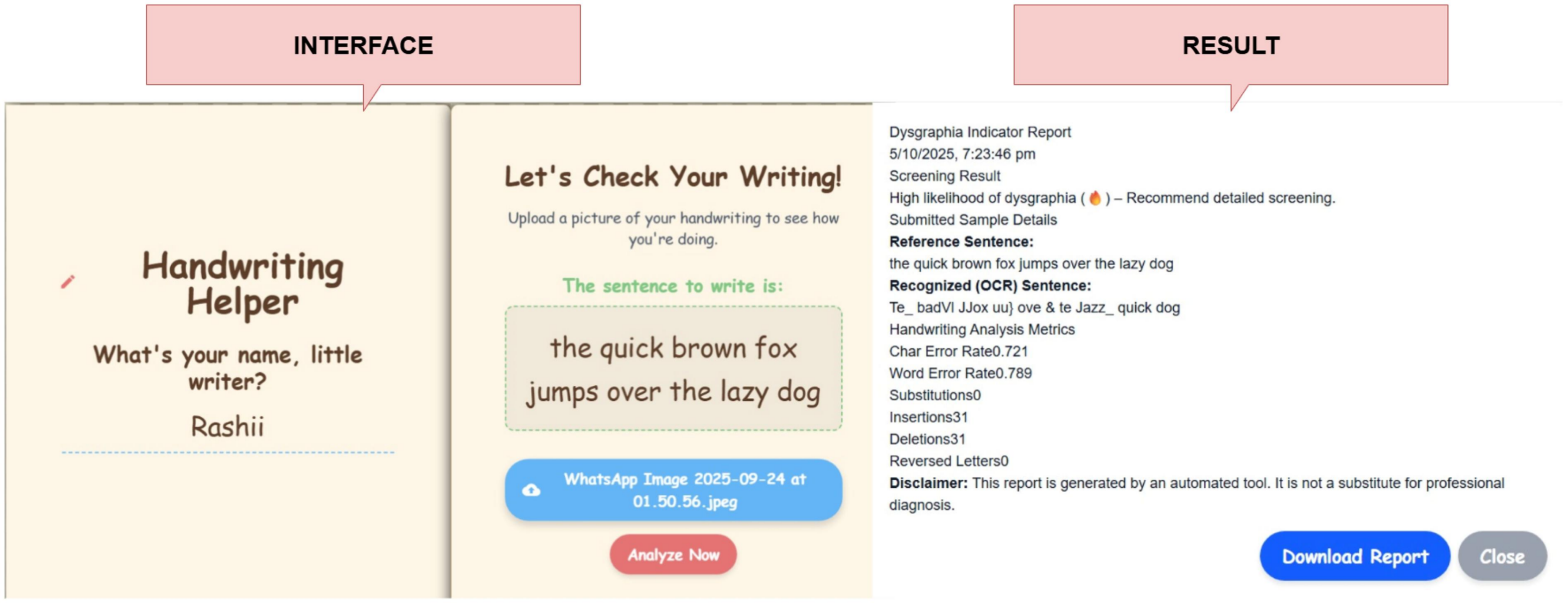
OCR tracker for handwriting analysis with its report shown.

A specialized software tool analyzes handwriting samples-whose expected text is known-using the EasyOCR library to extract the transcribed string from the input image. The resulting text is aligned with the ground-truth sentence via a sequence-matching algorithm to compute a suite of dyslexia-relevant metrics: Character Error Rate (CER), Word Error Rate (WER), and counts of substitutions, insertions, and deletions Figure 9. Additionally, the system identifies and tallies letter-reversal errors (e.g., “b” vs. “d”), which are distinctive markers of dyslexic writing. Threshold-based decision logic then classifies samples as “likely dyslexic” when one or more metrics exceed empirically determined cutoffs, flagging the writing for formal follow-up evaluation.

#### 4.1.3 Behavioral quiz outcomes

**Figure 10:**
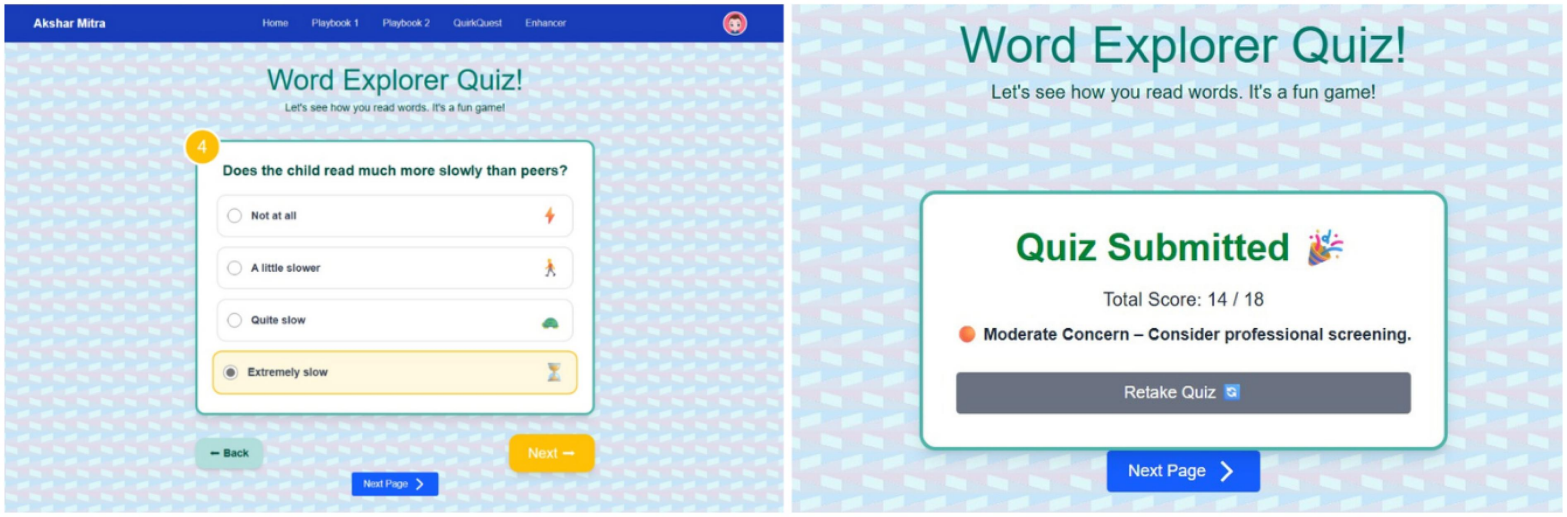
Screening quiz along with the results.

After the participant completes all questionnaire items Figure 10, the total Likert-scale score is computed and mapped to one of four risk categories: *Unlikely Dyslexia* (0–6), *Mild Signs* (7–13), *Moderate Concern* (14–20), or *High Concern* (21–27). The interface then displays the numeric score alongside its categorical label, enabling immediate, interpretable feedback.

#### 4.1.4 Reading support tool

**Figure 11:**
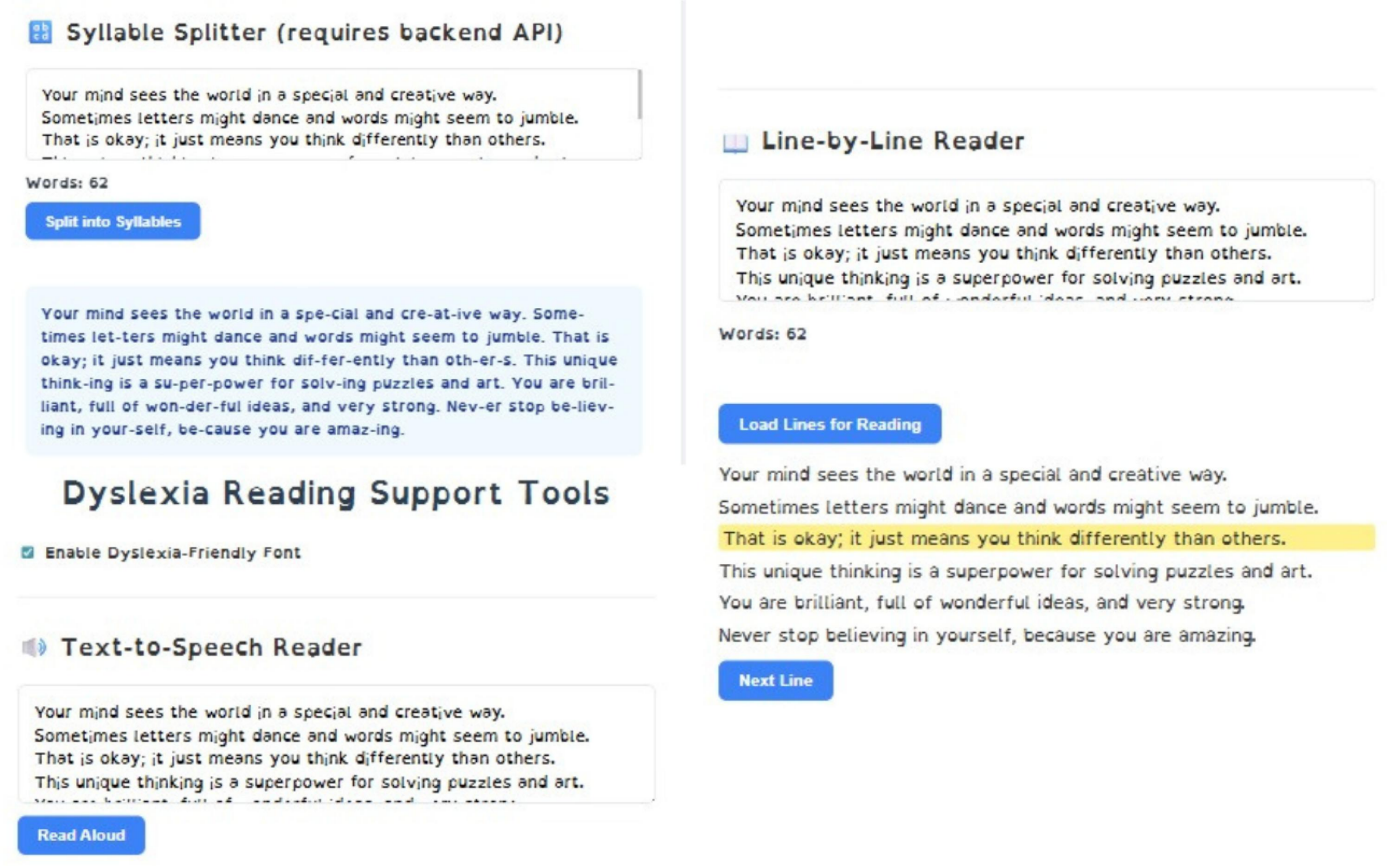
Reading supporting tool comprising of syllable splitter, text highlighter, text-to-speech reader.

A novel software application tailored for dyslexic readers synergistically combines three evidence-based support strategies. First, an integrated Text-to-Speech (TTS) engine vocalizes each word, reinforcing grapheme–phoneme correspondence and aiding auditory processing. Second, a line-by-line reading mode dynamically highlights one text line at a time, minimizing visual tracking errors and enhancing reading flow. Third, an auto-syllabification module segments longer words into pronounceable syllable units, reducing decoding effort and cognitive load Figure 11. By simultaneously targeting auditory, visual, and decoding deficits, the system provides a comprehensive assistive tool that empowers dyslexic individuals to improve reading accuracy and confidence.

#### 4.1.5 Gamified learning outcomes

**Figure 12:**
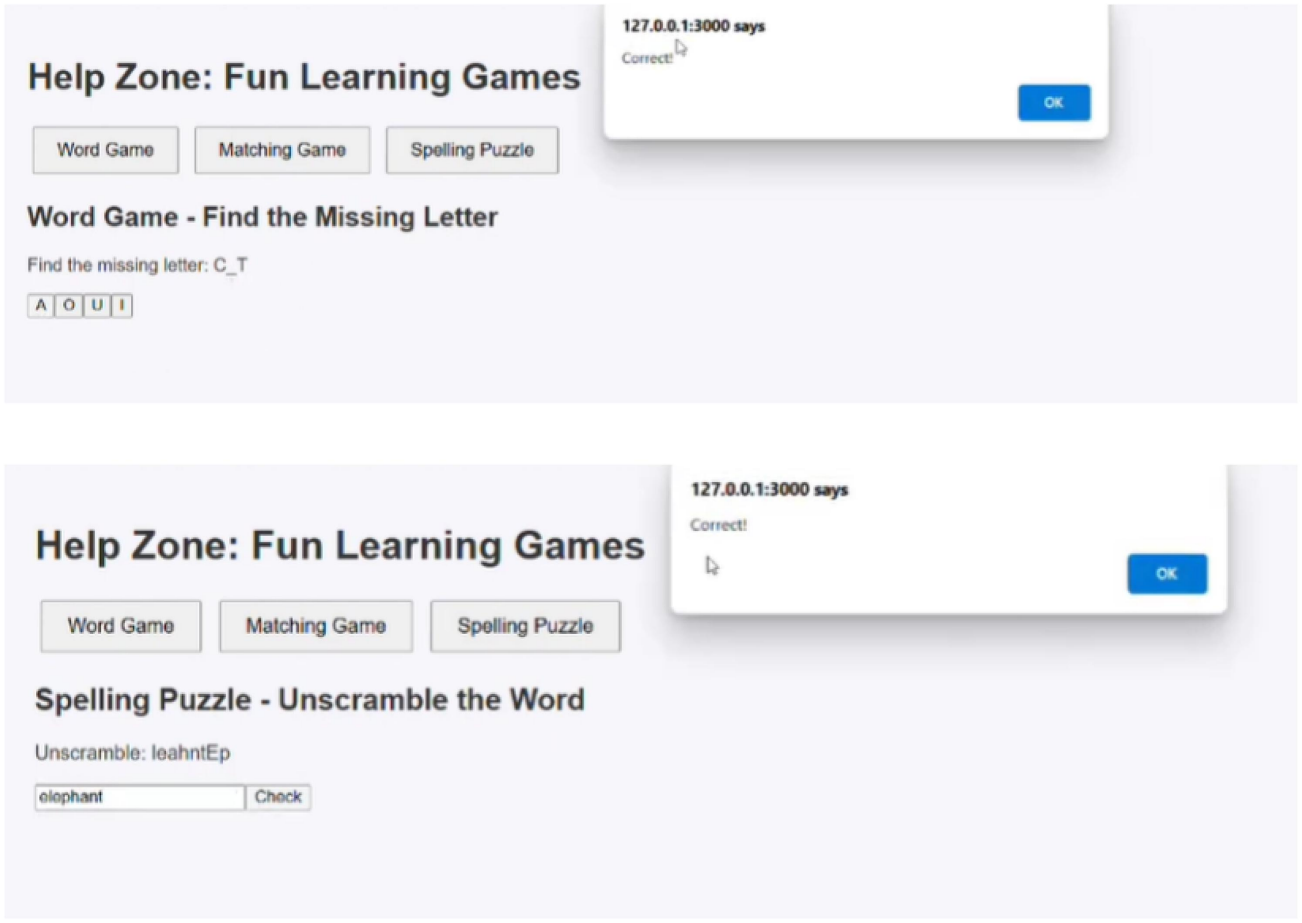
Gamified exercises.

The platform includes interactive games that promote letter–sound recognition and word formation skills. In the “Missing Letter” activity, children learn to identify letters within words using contextual image clues, enhancing phonemic awareness. The “Word Unscramble” game reinforces spelling and vocabulary by requiring users to reconstruct jumbled words Figure 12. These modules are both educational and diagnostic, capturing subtle learning patterns that support dyslexia assessment.

## 5 Conclusion

This study introduces a multimodal, technology-assisted framework for the early screening of dyslexia in children, combining eye-movement analysis, handwriting pattern recognition, and audio-based linguistic assessment within an adaptive, interactive environment. The proposed system employs real-time data acquisition and intelligent decision modules to identify early indicators of reading and cognitive difficulties. By unifying behavioral and linguistic modalities, the framework demonstrates the feasibility of data-driven, user-centered screening that can complement conventional diagnostic approaches and empower educators and caregivers with actionable insights.

Nevertheless, the study remains preliminary, as validation is currently constrained by small-scale datasets and limited participant diversity. Future work will focus on large-scale empirical studies (24), cross-linguistic adaptation, and the integration of explainable AI techniques to enhance interpretability and clinical trust. Strengthening ethical safeguards and data transparency will also be central to ensuring responsible deployment. Despite these constraints, this research establishes a meaningful foundation for developing intelligent, inclusive, and ethically aligned assistive technologies that bridge the gap between artificial intelligence and human-centered educational support for children at risk of dyslexia.

## Data Availability

The original contributions presented in the study are included in the article/Supplementary Material, further inquiries can be directed to the corresponding author.

## Appendix

### Quiz questions

The Behavioral Quiz module (Section 3.4) uses the following nine items. Respondents select one option per question on a 4-point scale (0–3).

1. Does your child tend to guess words instead of sounding them out while reading?
  - Never (0)
  - Every now and then (1)
  - Quite often (2)
  - Almost always (3)
2. Does your child find it hard to recognize common sight words (like “the,” “and,” “said”)?
  - Not at all (0)
  - Just a little (1)
  - Often (2)
  - All the time (3)
3. When reading aloud, does your child pause frequently or skip over words or even whole lines?
  - Not really (0)
  - Sometimes (1)
  - Quite frequently (2)
  - Very often (3)
4. Does your child mix up letters like “b” and “d” or “p” and “q”?
  - Rarely (0)
  - Occasionally (1)
  - Frequently (2)
  - Almost always (3)
5. Does your child misspell simple words in ways that seem unpredictable or inconsistent?
  - No (0)
  - Sometimes (1)
  - Often (2)
  - Very often (3)
6. Does your child find it difficult to rhyme words or pick out individual sounds in words?
  - Not at all (0)
  - A little bit (1)
  - Quite a bit (2)
  - A lot (3)
7. Does your child sometimes mix up sounds or syllables when speaking?
  - Not really (0)
  - Rarely (1)
  - Sometimes (2)
  - Often (3)
8. Does your child struggle to remember things that come in a sequence, like the alphabet or days of the week?
  - No (0)
  - Occasionally (1)
  - Often (2)
  - Very often (3)
9. Does your child have a hard time staying focused when reading or writing?
  - No (0)
  - Occasionally (1)
  - Frequently (2)
  - Almost constantly (3)
